# Lessons on data-informed decision-making for life-saving commodities in developing countries

**DOI:** 10.7189/jogh.11.03093

**Published:** 2021-08-07

**Authors:** Bennett Nemser, Blerta Maliqi

**Affiliations:** 1 University of the Western Cape, Cape Town, South Africa; 2 World Health Organization, Geneva, Switzerland

Health information is the foundation of public health, as it innervates decision-making within the functions and building blocks of the health system (eg, governance, financing, medical products/technologies, service delivery) [1]. Data-informed decision-making (DIDM) empowers stakeholders at any level of the health system – community, facility, sub-national, national or global – to identify problems and prioritize remedies during regular operation and emergencies [2]. Strengthening health systems – and ultimately achieving the Sustainable Development Goals (SDGs) – relies on equitable and timely access to life-saving commodities [1,3]. Based on experience implementing the recommendations of the UN Commission on Life-saving Commodities for Women and Children (UNCoLSC), we describe how commodity-related information systems and corresponding decision-making processes can be improved for more effective health care delivery during anticipated and unanticipated disruptions.

## DATA-INFORMED DECISION-MAKING

Health system performance depends on decisions by its stakeholders – from mothers deciding when and where to seek care; to clinicians deciding on appropriate treatments; to managers deciding when to procure commodities; to health policy makers deciding what to include in the essential medicines list. Optimizing and maintaining health system performance necessitates data-informed decision-making to continuously execute iterative cycles of problem identification, prioritized corrective action, and evaluation of results, and identification of new problems for the cycle to repeat. Moreover, timely DIDM supports continuous preparedness, which is critical when unforeseen external events occur, such as natural disasters, political unrest, or pandemics. COVID-19 has placed extraordinary pressure on supply chains and human resource capacity to deliver emergency and routine care. In rapidly changing settings, adaptive information systems with dynamic user data can enable quick DIDM to ameliorate bottlenecks and meet the vacillating needs of policy makers, managers, clinicians and users. Harnessing data for better strategic and operational decision-making is a vital lever for countries to reach and maintain universal health coverage in the face of any obstacle.

## LIFE-SAVING COMMODITIES FOR WOMEN AND CHILDREN

Annually, more than six million maternal, newborn, child and adolescent deaths occur – most of them preventable [3,4]. In 2012, the United Nations Commission on Life-saving Commodities for Women and Children (UNCoLSC) [5] outlined an ambitious agenda to broaden access to 13 high-impact, low-cost commodities to reduce preventable maternal, newborn and child health mortality (**Table 1**). To support this agenda, the Reproductive, Maternal, Newborn, and Child (RMNCH) Fund, a multi-UN agency funding mechanism, supplied short-term financial resources to 14 selected low- or middle-income countries (LMICs) for RMNCH acceleration plans. These plans were country-specific, developed by government-led coordinating mechanisms, and include a vast array of RMNCH-related activities (eg, staffing, training, commodity procurement, strengthening information systems, demand generation) [6]. Over five years, these countries experienced significant improvement in alleviation of systemic bottlenecks for life-saving commodities and stock availability [6].

**Table 1:** 13 Life-saving commodities

Commodities	Condition(s)
**Reproductive health**	
Female condom	Contraceptive
Implants	Contraceptive (long-term)
Emergency contraceptives	Contraceptive
**Maternal health**	
Oxytocin	Post-partum hemorrhage (PPH)
Misoprostol	Post-partum hemorrhage (PPH)
Magnesium sulfate	Eclampsia / Pre-eclampsia
**Newborn health**	
Injectable antibiotics	Bacterial infection, sepsis
Antenatal corticosteroids	Pre-term respiratory distress syndrome
Chlorhexidine	Newborn cord care
Resuscitation equipment	Newborn asphyxia
**Child health**	
Amoxicillin	Pneumonia
Oral rehydration salts (ORS)	Diarrhea
Zinc	Diarrhea

## IMPROVING DATA-INFORMED DECISION-MAKING FOR LIFE-SAVING COMMODITIES

Design and implementation of RMNCH acceleration plans, heavily relied on DIDM to strategically guide commodity programming and investment [6,7]. Across countries, commodity-related information systems and associated DIDM processes are heterogenous and face sizable challenges. The following DIDM life-saving commodities key areas have to be strengthened: 1) comprehensiveness of data types; 2) accessibility and transparency; 3) local adaptation; 4) inclusivity of decision makers; and 5) implementation and impact evaluation.

## COMPREHENSIVENESS OF DATA TYPES

Addressing any systemic health delivery issue in LMICs, such as access to and appropriate administration of life-saving commodities, requires a multitude of data types for DIDM. Rather than an exhaustive list, below are critical data types often requiring improvement across countries:

1. **Commodity forecast, pricing and procurement**: Generating accurate commodity procurement needs across government and partners as well as gaining timely access to pricing options for life-saving commodities strengthens a country’s cost-efficiency and purchasing power within the market. Unfortunately, RMNCH commodity pricing data in public domain is scarce, while information sharing among government and partners on demand quantification and procurement is infrequent or not coordinated for efficient purchasing practices.
2. **Supply management and distribution**: Tracking commodity shipment, receipt and status of availability at every location - from the central store to health facilities and community providers - helps ensure preparedness, efficient commodity usage, and loss reduction. However, generating this type of data, typically via electronic logistic management information systems (eLMIS), is hindered by uneven access to technology and connectivity, timeliness and quality of data entry and transfer, as well as its actual utilization across all facilities / providers.
3. **Quality of care**: Measuring the appropriateness, quality, and client perspectives of care delivered by providers allows assessment of clinical standards adherence and identification of areas for improvements. Measurement of quality of care is highly contextualized and resource intensive, which limits scope across services delivered and feasibility of standardized routine monitoring.
4. **Financial resource allocations**: Collating information on financial resources available from the government and all development partners can facilitate prioritization and alignment of planned commodity- and other RMNCH-related activities. To improve utility, disaggregated budget line items are essential; however, partners often resist sharing detailed budget data.
5. **Qualitative data on upstream systemic barriers**: Synthesized and nuanced qualitative policy, regulatory, and programmatic data helps understand where bottlenecks occur along the RMNCH programme implementation pathway (eg, MNCH Asset Tracker or RMNCH Situation Analysis [6]^6^). While these data exist in various forms and sources, time and resources are required to collate, manage and update this information for decision-making.

## ACCESSIBILITY AND TRANSPARENCY

Comprehensive data can only be useful if they are transparent and accessible to decision makers. Timely and effective course corrections often require more than only summarized data at national or regional level, so disaggregated data at health facility or community level are needed to take targeted action. While health management information systems (eg, District Health Information System 2, DHIS2) are increasingly more accessible at facility level, other critical data sets (eg, eLMIS) are often inaccessible to public health stakeholders. For example, DHIS2 can illustrate the number of children treated for a condition (eg, pneumonia) by health facility, but eLMIS data on commodity stockouts for the same facility is not accessible. To manage these complex, cross-organizational data systems, countries are progressively deploying data interoperability layers, which connect disparate information systems and allows easier accessibility by decision makers. For example, an interoperability layers could connect existing DHIS2, eLMIS and staffing information systems to allow decision makers to simultaneously view service delivery, commodity availability, and staffing levels for each health facility across the country.

**Figure:**
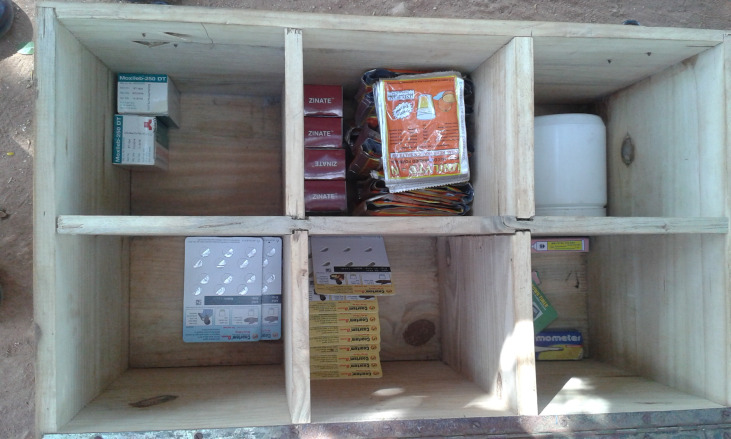
Photo: Medicine kit, including life-saving commodities, of a community health worker in Southern Malawi (from Bennett Nemser’s own collection, used with permission).

For transparency, expansive authorization opens data sets to more users, which can drive data quality improvements, hypothesis generation for further exploration, and collectively more effective DIDM [2]. Transparency is not limited to sharing between the government and development partners, but should extend to community members, public stakeholders and civil societies from local to global levels. Stakeholders on the far ends of the international development continuum - local beneficiaries (eg, women and children in developing countries) and funders (eg, taxpayers in developed countries) - often have the least access to data. Transparency and accessibility of data sources builds trust and more accurate learning amongst all stakeholders.

## LOCAL ADAPTATION

Data systems must be adapted to generate operationally meaningful information for local decision makers to address deficiencies in accessing and using life-saving commodities. Put simply, no two countries, communities, decision makers or decisions are exactly the same, so data systems should reflect this diversity. Two examples of this local adaptation include the RMNCH Scorecard and the Network for Improving Quality of Care (QOC) for Maternal, Newborn and Child Health. The RMNCH Scorecard approach relied on in-country stakeholders from multiple operating levels to generate a unique set of actionable indicators, which were transparent and publicly shared to illicit focused attention and corrective action in each locality. Second, the QOC Network [8] was designed to empower teams of local health staff to initiate short cycles of problem identification, data collection and evaluation to correct underperforming service delivery. These approaches are less rigid and place higher importance on indicators valued by end users for the unique challenges facing their local women and children.

## INCLUSIVITY OF DECISION-MAKERS

Prioritizing and addressing the most pressing problems in a community requires input from a diverse and inclusive set of stakeholders. While decisions are informed by data, the perspectives and biases – both positive and negative - of decision makers hold great influence. Too often, civil society and community leaders are left out of prioritization and decision-making processes for life-saving interventions that deeply impact their communities. For example, the Global Financing Facility (GFF) at its onset limited participation and influence of civil society, but quickly shifted to a more inclusive approach [9]. Without guidance from the most vulnerable stakeholders, development partners risk misdirecting projects and jeopardizing the value of supply chains and service delivery infrastructure.

## IMPLEMENTATION AND IMPACT EVALUATION

The final step for effective DIDM for life-saving commodities is evaluating the implementation and impact of decisions to learn for iterative improvements. The RMNCH Fund provided short-term catalytic funding for life-saving commodities, but the relatively short project cycle and budget only permitted an evaluation focused on implementation rather than impact [10]. While funds were efficiently deployed and activities effectively implemented by country teams, the impact of program decisions on health outcomes for women and children were unclear. Projects with a larger footprint and time horizon, such as the GFF, are wise to invest in a robust impact evaluation (with preferably rapid evaluation cycles and alongside implementation research) to fully understand the effectiveness of their decision-making on beneficiaries.

## CONCLUSION

Ensuring all women and children have access to life-saving commodities is a fundamental component of universal health coverage and has become deeply entrenched in the global health agenda [3]. Achieving these ambitious goals in each country will rely on the strength of commodity-related data and associated decision-making processes. Adaptive data systems – with commodities as a central element – can help countries build back better as the COVID pandemic wanes. Robust DIDM enables health professionals and community stakeholders to more accurately learn from the past and decide their future trajectory.
